# I don’t know where I’m going or where I come from. Self-disorders in schizophrenia.

**DOI:** 10.1192/j.eurpsy.2023.2271

**Published:** 2023-07-19

**Authors:** M. D. C. Vallecillo Adame, L. Rodríguez Andrés, C. de Andrés Lobo, T. Jimenez Aparicio, M. Queipo de Llano de la Viuda, G. Guerra Valera, A. A. Gonzaga Ramirez, M. Fernández Lozano, M. J. Mateos Sexmero, N. Navarro Barriga, B. Rodríguez Rodríguez, M. P. Pando Fernández, M. Calvo Valcárcel, P. Martínez Gimeno, M. A. Andreo Vidal, I. D. L. M. Santos Carrasco

**Affiliations:** 1 Hospital Clínico Universitario Valladolid, Valladolid; 2 Hospital Infanta Leonor, Madrid, Spain

## Abstract

**Introduction:**

In the early stages of schizophrenia the person experiences feelings of strangeness about themselves, difficulty in making sense of things and difficulty in interacting with their environment. Based on this, self-disorder assessment instruments have been developed and empirical studies have been conducted to assess people at risk of developing a schizophrenia spectrum disorder. These studies show that self-disorders are found in pre-psychotic stages and that their manifestation can predict the transition to schizophrenia spectrum disorders.

**Objectives:**

We present the case of a patient with multiple diagnoses and mainly dissociative symptoms who, after years of evolution, was diagnosed with schizophrenia.

**Methods:**

Bibliographic review including the latest articles in Pubmed about self-disorders and schizophrenia.

**Results:**

We present the clinical case of a 51-year-old woman with a long history of follow-up in mental health consultations and with multiple hospital admissions to the psychiatric unit, with several diagnoses including: dissociative disorder, histrionic personality disorder, adaptive disorder unspecified psychotic disorder and, finally, schizophrenia. The patient during the first hospital admissions showed a clinical picture of intense anxiety, disorientation and claiming to be a different person. The patient related these episodes to stressors she had experienced, and they improved markedly after a short period of hospital admission. Later, psychotic symptoms appeared in the form of auditory and visual hallucinations and delusional ideation, mainly of harm, so that after several years of follow-up and study in mental health consultations and in the psychiatric day hospital, she was diagnosed with schizophrenia and treatment with antipsychotics was introduced, with a marked clinical improvement being observed.

**Conclusions:**

It is important to take into account this type of symptoms (self-disorders), as they allow the identification of individuals in the early stages of the disorder and create the opportunity for early therapeutic interventions.

**Disclosure of Interest:**

None Declared

